# Chimpanzees recognize their own delayed self-image

**DOI:** 10.1098/rsos.170370

**Published:** 2017-08-09

**Authors:** Satoshi Hirata, Kohki Fuwa, Masako Myowa

**Affiliations:** 1 Wildlife Research Center, Kyoto University, Kyoto, Kyoto 606-8203, Japan; 2 Earth Mate Chimpanzee Next, Tamano, Okayama 706-0316, Japan; 3 Graduate School of Education, Kyoto University, Kyoto, Kyoto 606-8317, Japan

**Keywords:** self-recognition, chimpanzee, delayed contingency, self-concept

## Abstract

Unlike mirror self-recognition, recognizing one's own image in delayed video footage may indicate the presence of a concept of self that extends across time and space. While humans typically show this ability around 4 years of age, it is unknown whether this capacity is found in non-human animals. In this study, chimpanzees performed a modified version of the mark test to investigate whether chimpanzees could remove stickers placed on the face and head while watching live and delayed video images. The results showed that three of five chimpanzees consistently removed the mark in delayed-viewing conditions, while they removed the stickers much less frequently in control video conditions which lacked a link to their current state. These findings suggest that chimpanzees, like human children at the age of 4 years and more, can comprehend temporal dissociation in their concept of self.

## 1. Introduction

The ability to recognize one's own appearance is assumed to be a proxy for complex psychological processes such as self-awareness and the concept of self [1,2]. Human infants begin to show self-recognition in mirrors from 18 to 24 months but do not exhibit self-recognition in delayed video images for another 2 years [3–7]. Previous works have debated the developmental asynchrony of live and delayed self-recognition. One theory holds that while self-recognition in a mirror requires a self-concept limited to the present time and location, delayed recognition requires a ‘self-concept proper’ in which one's sense of identity extends across time and space, which develops somewhat later than the ability for live self-recognition [8]. An extension of this theory is that delayed self-recognition is linked to autobiographical memory and that both delayed self-recognition and manifestation of autobiographical memory develop at almost the same time in human children, at around 4 years of age [8]. However, there has been no evidence to confirm this theory, and factors that are not specific to self-recognition might be involved in this developmental asynchrony [5,9]. In addition, a similar concept of developmental asynchrony has been reported between self-recognition in mirrors and live videos [10], and more data are warranted to prove this theory.

From a phylogenetic perspective, chimpanzees and other great apes have been shown to exhibit self-recognition in mirror studies [11–17], and this phenomenon has been reported in dolphins and elephants [18,19]. However, there is little evidence regarding whether animals can recognize themselves in images from different time periods. One study found that monkeys spent more time looking at live versus delayed video images of themselves [20]. Gorillas were also observed to show more interest in live videos compared with images recorded the day before [21]. Chimpanzees were able to discriminate between cursor movements on a computer monitor caused by real-time self-action and those caused by previously recorded self-action [22]. However, differences in viewing time and the ability to discriminate between images do not necessarily imply self-recognition. Thus, there is no clear evidence that recognition of delayed self-image occurred in these studies. This study used a modified version of the classic mark test to determine whether chimpanzees could remove stickers placed on their face and head while watching live and delayed video images. One study reported that 3-year-old human children, during the transition from live self-recognition ability to the capacity for delayed self-recognition, do not remove stickers from the head while viewing 2 s delayed images of themselves, but do so while watching live or 1 s delayed self-images [6]. That study on children indicates that comprehending a few seconds' delay is a critical factor for developing self-recognition. In this study, we tested chimpanzees' reactions to live and 1–4 s delayed images along with additional control videos, which lacked a link to their current state.

The aim of this study was to explore the reactions of chimpanzees to their delayed self-image. We tested five chimpanzees in experimental sessions beginning with the placement of 10 stickers on the head or face of a fully awake chimpanzee. Following a 2 min observation period during which no image was shown on the monitor, one of eight types of video was then presented on the monitor for 2 min. These included: a live self-video; a 1 s delayed self-video; a 2 s delayed self-video; a 4 s delayed self-video; a distant past video (more than one week old) showing the subject with stickers on the face and head; a distant past video (more than one week old) of the subject with no stickers on the face or head; a video of a human with stickers on the face and head; and a video of a human with no stickers on the face or head. Each chimpanzee watched five repeats of each of the eight conditions.

## 2. Material and methods

### 2.1. Participants

Five chimpanzees (*Pan troglodytes*) participated in this study. All subjects lived in a group together at the Great Ape Research Institute, Hayashibara Biomedical Laboratories, Inc., Japan, in an enriched environment consisting of a 7400 m^2^ outdoor enclosure and several indoor areas [23].The group consisted of Loi (male, 10 years old), Zamba (male, 10 years old), Tsubaki (female, 10 years old), Mizuki (female, 9 years old) and Misaki (female, 7 years old; note that these data refer to age at the start of the test period). Mizuki was raised by human carers from a few days after birth at another biomedical institute. Since arriving at the Great Ape Research Institute (at age 2 years and 1 month), she has spent the majority of her time with other chimpanzees in outdoor and indoor compounds. Loi, Zamba, Tsubaki and Misaki were mother-reared at another biomedical institute until they were about 3 years old, after which they moved to the Great Ape Research Institute and spent time together in outdoor and indoor compounds. All of these chimpanzees had had extensive interaction with human researchers, including sleeping together in their night room and undergoing previous behavioural cognitive experiments [23]. Various fruits and vegetables were provided 3–10 times a day. Water was freely available, and the chimpanzees were never food-deprived. The research protocol was approved by the Animal Welfare and Animal Care Committee of Hayashibara Great Ape Research Institute (GARI-051101).

### 2.2. Observations and pretests prior to the experiment

While a formal test for mirror self-recognition was not conducted until the pretest period of the present experiment described below, it should be noted that all chimpanzees were exposed to a mobile mirror during their daily interactions with human researchers for environmental enrichment. In addition, one of their indoor rooms was equipped with a mirror positioned on the wall and there was also a reflective stainless steel surface in the playground that could be used as a mirror. Systematic data were not collected on chimpanzees' prior responses to mirrors and reflective surfaces. However, researchers and caretakers had observed on multiple occasions that the three chimpanzees who exhibited self-recognition in live and delayed tests in this study (Loi, Tsubaki and Mizuki) had all looked at mirrors and reflective surfaces and made self-directed gestures, such as opening the mouth and looking into it. The other two individuals (Zamba and Misaki) were never observed using mirrors or reflective surfaces in this way prior to the current test.

Before the delayed video testing, one pretest session involving mirror exposure and three pretest sessions involving live video exposure were conducted. An experimental room that was hexagonal in cross-section, 7.6 m^2^ in area and 5 m tall, was used for these sessions. The pretest was conducted to confirm whether chimpanzees could recognize their own mirror image. Loi and Zamba both entered the experimental room by themselves. Tsubaki was accompanied by her dependent infant. Mizuki and Misaki were not related, but had a close bond and came together to the experimental room. This pair also gave birth to their baby during the course of the experiment, who subsequently accompanied them. Familiar experimenters (S.H. and K.F.) stayed in the same room with the chimpanzees during testing. Additional staff were occasionally in the room, typically to take care of the infants who were not being tested.

The pretest session involved three main steps. In step 1, four circular stickers (silver and 8 mm in diameter) were placed on areas directly visible to the chimpanzee (i.e. one sticker per arm and leg). Responses were then observed for about 30 s. In step 2, 10 circular stickers were placed on the chimpanzee's face and head. Based on the close and long-term relationships between the chimpanzees and the researchers, the five chimpanzees accepted placement of stickers without apparent distress. In contrast with many previous studies using the mark test [1], we did not anaesthetize the chimpanzees or surreptitiously place stickers on them, because it has been shown that these factors are not crucial components of the self-recognition test [12]. We also felt that surreptitious placement could not be ensured during the repetitive implementation of the tests and repetitive anaesthesia for the purpose of this study would have been inappropriate. After the placement of stickers, each chimpanzee's spontaneous behaviour towards their own bodies was observed for 2 min without exposure to the mirror. To ensure that the chimpanzee remained in a relaxed state in their location to enable recording of their behaviour, an experimenter gave a small piece of food about every 20 s during this period. Receiving food during cognitive testing is a routine procedure for chimpanzees, and provides motivation to participate in experiments, although participation in testing was voluntary and they could obtain meals even if they did not take part. In step 3, after 2 min had elapsed, a 30 × 40 cm mirror was placed in front of the chimpanzee and their behaviour was again observed for 2 min while food was offered approximately every 20 s, regardless of the chimpanzee's behaviour during the test.

Three pretest sessions involving live video exposure were conducted. The aim of this pretest was to habituate the chimpanzees to the presence of a monitor and to provide experimenters with an opportunity to practise the delayed video testing procedures described below. In addition, these video images were used for the sessions of the delayed test for conditions 5 and 6 (see below). A video camera was placed at the top of the 19-inch monitor that presented the live video footage, and filmed an area from slightly below the chin to slightly above the top of the head. This camera was one of the two cameras used to record all testing sessions. The image on the monitor was flipped from the normal orientation, to ensure that the left–right correspondence was the same as that of a mirror image. The three-step procedure for this video pretest was the same as the pretest involving the mirror, except that the chimpanzee's image was displayed on a monitor rather than a mirror. An experimenter gave a small piece of food in the same manner as in the mirror exposure test. The experimenter stayed outside of the range covered by the video camera but their hand become visible on the monitor when food was given to the chimpanzee.

### 2.3. Delayed video testing

**Figure 1.**
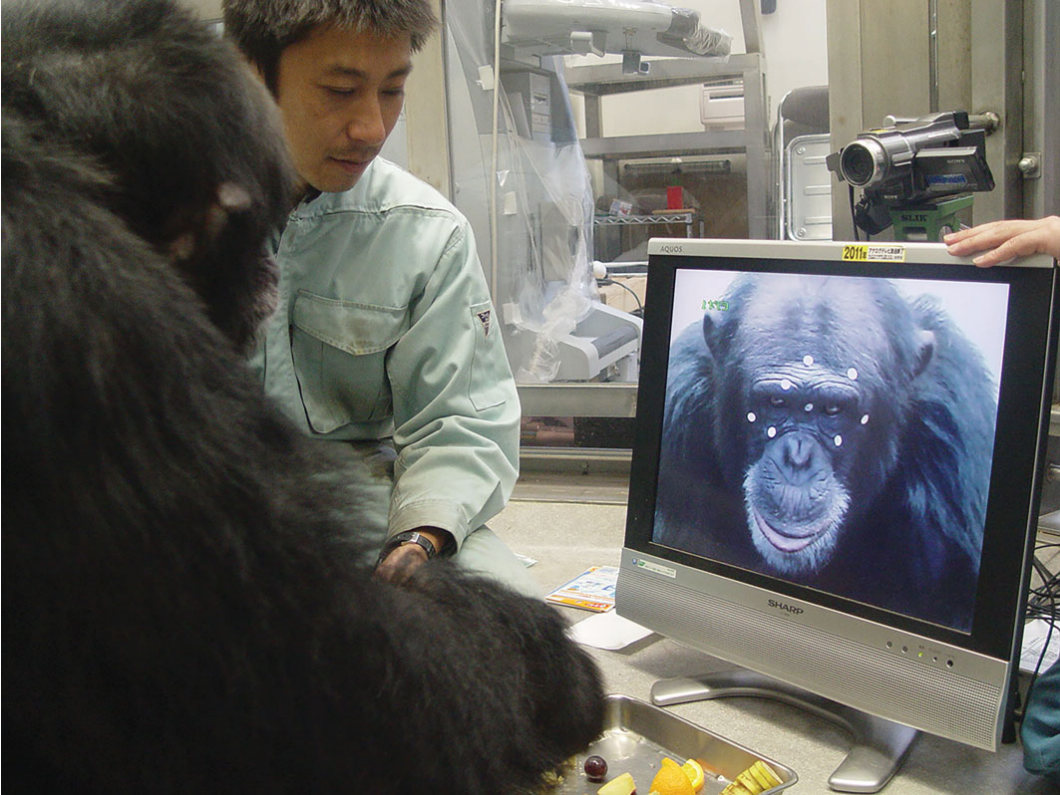
Experimental set-up. A male chimpanzee faces a monitor. Stickers are placed on his face and head.

The test was conducted in the same indoor experimental room, and familiar experimenters (S.H. and K.F.) stayed in the same room with the chimpanzees during testing (figure 1). In step 1, one of the experimenters placed 10 circular stickers (silver, 8 mm in diameter) on the chimpanzee's face and head. After sticker placement, each chimpanzee's spontaneous behaviour was observed for 2 min during which no images were shown on the 19-inch monitor. A video camera was placed at the top of the monitor. The camera recorded a view of the area from slightly below the chin to slightly above the top of the head of the chimpanzee. There was a second camera that recorded the entire testing area.

In step 2, one of the following eight videos (conditions 1–8) was presented for 2 min: a live video (condition 1), a 1 s delayed video (condition 2), a 2 s delayed video (condition 3), a 4 s delayed video (condition 4), a video of the subject that was filmed more than one week prior in which stickers had been placed on the face and head of the subject (condition 5), a video of the subject that was filmed more than one week prior, in which no stickers were present on the face and head (condition 6), a video of a human who had stickers placed on the face and head (condition 7) or a video of a human with no stickers (condition 8). In steps 1 and 2, an experimenter gave a small piece of food about every 20 s to ensure that the chimpanzee remained in a relaxed state to enable recording of their behaviour, regardless of the chimpanzee's behaviour during the test.

For the live video condition, the image filmed by the camera placed at the top of the monitor (described above) was presented on the monitor. The image on the monitor was flipped from the normal orientation to ensure that the image shown was the same as that of a mirror image. The 1 s, 2 s and 4 s delayed conditions were created using a special device (VM-700, ITO Co. Ltd., Osaka, Japan) that was plugged into the same video camera. For conditions 5 and 6 in which the delay exceeded 1 week, we edited video scenes recorded during the prior testing and/or pretest involving live video exposure. For condition 5, we edited the video to include only images of the chimpanzee in which (i) there were more than three stickers on their face and head, and (ii) no self-directed behaviour was evident to ensure there was no coincidental matching of the subject's current self-directed behaviour to that of the pre-recorded video. For condition 6, we edited the video to ensure that there were no stickers nor any self-directed behaviour. For the human videos, we recorded a person with whom the subjects were familiar but who was not present during testing. In both human videos, the models engaged in slight, natural movements so that the video was not a still image, but no self-directed behaviour occurred. The only difference between the two human videos was the presence (condition 7) or absence (condition 8) of 10 stickers located upon the head and face.

Each session contained two conditions, each starting from step 1. We conducted 20 sessions for each of the five chimpanzees; thus, there were 40 conditions and five repeats of each of the eight conditions. The order of conditions was counterbalanced. The sessions were separated by at least one week, with an average of one month to avoid habituation and maintain the subjects' interest. The entire test (20 sessions) was carried out across 2 years and 2 months.

### 2.4. Analysis

The following behaviours were coded from the video recordings: (1) removal of stickers, (2) self-directed behaviour toward the head or face (i.e. the subject touching the face or head with their hand), (3) contingency checking behaviour, such as opening the mouth and shaking the hands and (4) looking at the monitor. Notably, (i) the removal of stickers is not mutually exclusive with self-directed behaviour, and the former requires the latter and (ii) the coding of whether a subject was watching the monitor overlaps with other coded behaviours. Specifically, the coding included: ‘removal of the sticker while watching the monitor’, ‘removal of the sticker while not watching the monitor’, ‘self-directed behaviour while watching the monitor’, ‘self-directed behaviour while not watching the monitor’, plus contingency checking behaviour.

The number of stickers on a subject's face and head at the start of the test (i.e. when the monitor was switched on) varied, as some of the 10 stickers originally placed were removed by subjects' groping behaviour. Therefore, we calculated the proportion of the number of stickers that were removed during the test by dividing the number of stickers removed during the test by the number of stickers at the start of the test, and compared these proportions across experimental conditions. We continued recording each subject's behaviour for 2 min after the start of the test, but the chimpanzees occasionally removed all of the stickers before the 2 min had elapsed. In such cases, we considered the time until the subject removed the last sticker as the test period, and calculated the proportion of the duration of a particular behaviour (e.g. self-directed behaviour while watching the monitor) by dividing the duration of that behaviour by this adjusted test period.

A rater who was unaware of the experimental conditions and hypotheses coded all of the video recordings. A second rater coded 20% of the sessions to measure inter-rater reliability. Cohen's *κ* for the above-mentioned four mutually exclusive coding categories was 0.813 (*p* < 0.001). The statistical tests were non-parametric as we could not assume normality in the dataset. Data from each individual was treated separately because there were individual differences in responses.

## 3. Results

### 3.1. Pretests with mirror and live video exposure

Three chimpanzees (Loi, Tsubaki and Mizuki) removed all of the stickers while watching mirror and live video images during the pretests. The remaining two individuals (Zamba and Misaki) did not remove any stickers placed on their face and head during the mirror and live video exposure pretests, but they removed all of the stickers placed on the visible parts of the body such as the arms and legs.

### 3.2. Delayed video testing

**Figure 2.**
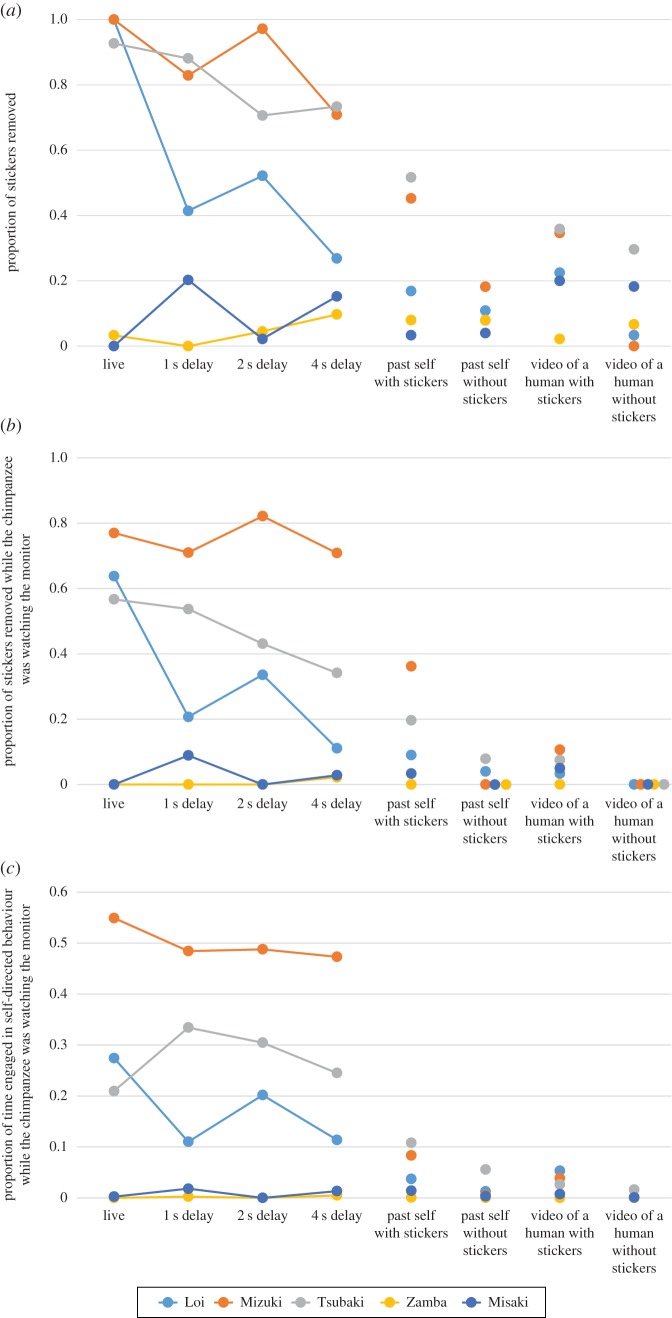
Responses of chimpanzees to video conditions. Each data point represents the average of five trials. (*a*) Proportion of stickers removed during the test period across different experimental conditions. (*b*) Proportion of stickers removed while the chimpanzee was watching the monitor across different experimental conditions. (*c*) Proportion of time engaged in self-directed behaviour while the chimpanzee was watching the monitor across different experimental conditions.

The proportion of stickers removed across the eight conditions among the five subject chimpanzees was not significantly different on the group level (Friedman's test, *p* = 0.36). However, there were statistically significant differences in the proportion of stickers removed while watching the monitor across the eight conditions (Friedman's test, *p* = 0.008) and the proportion of time spent engaging in self-directed behaviour while watching the monitor across the eight conditions (Friedman's test, *p* = 0.018) on the group level (figure 2). Because there were large individual differences in the chimpanzees' responses, below we describe the results further on an individual level.

Three of five chimpanzees (Loi, Tsubaki and Mizuki) consistently engaged in self-directed behaviours, exhibiting hand/finger actions directed to their own face and head, and removing the stickers while watching the image on the monitor in the live, 1 s, 2 s and 4 s delay conditions (conditions 1–4; electronic supplementary material, movie S1, S2). The responses of these three individuals were significantly different across the eight conditions according to the following three measures: the proportion of stickers removed (Kruskal–Wallis test, Loi, *H* = 20.43, *p* < 0.01; Tsubaki, *H* = 25.68, *p* < 0.01; Mizuki, *H* = 25.08, *p* < 0.01; figure 2*a*), the proportion of stickers removed while watching the monitor (Loi, *H* = 20.86, *p* < 0.01; Tsubaki, *H* = 21.57, *p* < 0.01; Mizuki, *H* = 24.88, *p* < 0.01; figure 2*b*) and the proportion of time spent engaging in self-directed behaviour while watching the monitor (Loi, *H* = 21.54, *p* < 0.01; Tsubaki, *H* = 23.61, *p* < 0.01; Mizuki, *H* = 29.88, *p* < 0.01; figure 2*c*).

Conditions 5–8 were used to rule out the possibility that the self-directed behaviour we observed while chimpanzees watched the monitor would emerge when viewing any stimuli on the monitor (condition 8), mere appearance of stickers in the image shown on the monitor (condition 7), a self-image video with no stickers (condition 6), or a self-image video without relevance to their current state (condition 5). The proportion of time spent on self-directed behaviour while watching the monitor was not different between the live and briefly delayed conditions (1–4 s; Fligner–Wolfe's test, Loi, FW = 135, *p* = 0.053; Tsubaki, FW = 50, *p* = 0.87; Mizuki, FW = 155, *p* = 0.87), whereas the corresponding proportions of self-directed behaviour were significantly lower in conditions 5–8 (Fligner–Wolfe's test, Loi, FW = 210, *p* < 0.001; Tsubaki, FW = 223, *p* = 0.009; Mizuki, FW = 210, *p* < 0.001).

One of the three chimpanzees who engaged in self-directed behaviour, Mizuki, exhibited contingency checking in the early stages of testing during trials in which past-self videos were shown (electronic supplementary material, movie S3). The other two chimpanzees, Loi and Tsubaki, did not exhibit contingency checking behaviour, but did engage in several self-directed behaviours while watching the videos for conditions 5–8.

To test for the possible effects of learning during the course of the experiment, we examined responses across sessions. The result showed no statistically significant increases or decreases in the three measures as a function of session in (i) the proportion of stickers removed, (ii) the proportion of time spent engaging in self-directed behaviour while watching the monitor, and (iii) the proportion of stickers removed while watching the monitor (Jonckheere–Terpstra's test, *p* > 0.1 for all of the individuals). Moreover, the three chimpanzees who exhibited evidence of recognition while watching delayed videos each removed one or more stickers during the first presentations (4 s delay in the case of Loi and 2 s delay in the case of Tsubaki and Mizuki; the order of conditions was varied across subjects to counterbalance among individuals).

## 4. Discussion

Our study found that three of the chimpanzees tested consistently removed the stickers in delayed-viewing conditions. The current procedure differed from the classic mark test in that we did not place stickers surreptitiously under anaesthesia or during play [1,14]. Instead, we placed stickers non-surreptitiously while the chimpanzees were awake, in line with reports that the surreptitious placement of marks is not critical for investigating self-image recognition in the mark test in chimpanzees [12]. In the classic mark test, the frequency of self-directed behaviour is compared with that of a baseline period before the subject has viewed self-images. By contrast, we compared the frequency of self-directed behaviour across eight conditions. Four of the eight conditions (conditions 5–8) served as a baseline, in the sense that self-directed behaviours in these conditions were elicited without the subject recognizing the link between the image on the monitor and the current state of the self. The results revealed that self-directed behaviour was more frequent in the live and 1–4 s delay conditions than in the other conditions, demonstrating that these three subjects were able to discriminate whether the image shown reflected the current state of the self or not. In other words, our results suggest that the three chimpanzees were capable of self-recognition in live and delayed self-images.

The contingency checking behaviour of one of the chimpanzees, Mizuki, during trials in which past-self videos were shown suggests that she was actively probing the presence or absence of contingency while she was watching the video. The other two chimpanzees that removed stickers consistently, Loi and Tsubaki, did not exhibit contingency checking behaviour, but did engage in several self-directed behaviours while watching the videos for conditions 5–8. These self-directed behaviours probably facilitated the subjects' recognition of the lack of contingency when they were watching videos that were more than one week old in conditions 5 and 6, as the self-directed behaviours were removed from these videos. Previous studies have shown that contingency checking behaviour is a possible learning mechanism underlying successful performance in mirror self-recognition by apes [12] and human children [24]. In addition, capuchin monkeys were able to detect contingency but did not engage in active contingency checking behaviour nor showed evidence of mirror self-recognition [20,25]. Therefore, the emergence of contingency checking behaviour can be considered as an important sign of self-recognition in mirror or video images from both ontogenetic and phylogenetic perspectives.

Our analysis of possible changes in the chimpanzees' behavioural responses during the entire period of study (over more than 2 years) did not identify any trends in recognition. The three chimpanzees exhibited evidence of recognition while watching delayed videos during the first presentations, indicating that the chimpanzees were able to recognize their self-image in the delayed video conditions from the beginning of the experiments.

We have thus far discussed the positive results from three of five chimpanzees. The other two chimpanzees exhibited almost no self-directed behaviour or removal of stickers placed on the head and face (figure 2). Importantly, this pattern is consistent with most prior mirror-recognition tests involving chimpanzees, in which reported passing rates typically vary between 20% and 80% [1,15–17].

While the interpretation of these negative results remains unclear, individual variation in the capacity for self-recognition is possible and motivational factors may also play a role. However, the two chimpanzees easily removed the visible stickers that were placed on their arms and legs. Therefore, the fact that these two individuals did not remove the stickers that were placed on non-visible parts of their face cannot be ascribed to simple lack of motivation to remove the stickers. There may be individual variations in the depth of the level of self-recognition in chimpanzees. Alternatively, subtle differences in the rearing environment may have led to the varied performance in this task. A cross-country study of human children found cultural differences in their mirror self-recognition [26]. Thus, the fact that experience and other environmental factors affect self-recognition may be common to chimpanzees and humans.

Researchers have reported similarities in various types of higher cognitive functioning between chimpanzees and humans, such as memory, tool-use and symbolic representation [27]. The present findings extend our understanding of the capacity for self-recognition in chimpanzees and humans, suggesting that both species can comprehend temporal dissociation in their concept of self. In one study with human children, a 3-year-old boy was reported to say ‘This is my friend, isn't it’, after watching a 2 s delay video of himself. This indicates that the boy was unable to recognize himself when the video was delayed by 2 s, even though he was able to recognize himself in a mirror [6]. This example indicates that temporal matching plays an important role in visual self-recognition of children under 4 years of age and that inserting a brief delay disrupts their self-recognition. Children begin to understand delayed self-image at around 4 years of age, at which point they are generally able to recognize themselves over time and project themselves into the past. This ability has previously been proposed to be uniquely human [28].

There may be criticism against our study, as our method was different from the classic mark test and the subject chimpanzees knew that the stickers were being placed. A future direction of study would be to use the classic mark test paradigm, which requires surreptitious placement of a marker/sticker, to investigate delayed self-recognition in non-human animals. However, this approach would face the following two challenges: first, it would be difficult to secure a sufficiently large number of subjects. Surreptitious placement of a marker/sticker is ideally accomplished as a single trial, as has been seen in studies with human children that used between-subject comparisons to test live versus delayed self-recognition [3–7]. To conduct between-subject comparison across live versus multiple delayed conditions, a large number of subjects are needed, particularly considering the fact that not all chimpanzees displayed self-recognition, even in the live condition. Second, if within-subject comparisons are used instead of between-subject comparison, then repetitive surreptitious placement of a marker/sticker to the same subject is necessary. This is not an easy task, as the likelihood of failure to place the marker surreptitiously (i.e. the chimpanzee notices the placement of the marker) increases with repetition.

Furthermore, if such a study relied on using dye that cannot be removed easily as a marker (as in the case of original study with chimpanzees [1]), chimpanzees may become habituated to the presence of dye that cannot be removed. Thus, they may become less motivated to engage in self-directed behaviours to remove the dye. This is the reason that we used removable stickers in our study; that is, we felt that surreptitious placement of stickers could not be ensured during repetitive trials using the methods previously established. We acknowledge that verification of our modified version of the mark test is warranted, and a novel technique is necessary to overcome the challenges described above to apply the classic mark test for delayed condition. With that said, some evidence suggests that surreptitious marking or anaesthesia is not a necessary condition for testing self-recognition. Fully informing the subjects about the placement of a mark did not enhance performance in a mirror self-recognition task in human infants [12]. Monkeys who were trained to touch a mark in an operant conditioning paradigm still failed to pass the mark test for mirror self-recognition [29]. Our data were consistent with these findings. That is, the three chimpanzees that showed positive evidence in the delayed condition also showed positive evidence (i.e. removal of stickers) in all of the mirror and live video conditions that were conducted as pretests. As suggested by Bard *et al*. [12], we consider that overt marking, without the need for anaesthesia and isolation at testing, is a useful method for future comparative and developmental studies.

Another future direction of study would be to extend the duration of the delay to match that of most of the human studies [4,5,7,9]. As our study is the first systematic observation of chimpanzees' reaction to delayed self-images, further research using different approaches will be necessary to validate our findings.

## Data accessibility

Data used in this article are available on Dryad Digital Repository (http://dx.doi.org/10.5061/dryad.2g5r0) [30].

## References

[1] Gallup GG Jr 1970 Chimpanzees: self-recognition. Science 167, 86–87. (doi:10.1126/science.167.3914.86) 4982211 10.1126/science.167.3914.86

[2] Suddendorf T, Butler DL 2013 The nature of visual self-recognition. Trends Cogn. Sci. 17, 121–127. (doi:10.1016/j.tics.2013.01.004) 23410584 10.1016/j.tics.2013.01.004

[3] Amsterdam B 1972 Mirror self-image reactions before age two. Dev. Psychobiol. 5, 297–305. (doi:10.1002/dev.420050403) 4679817 10.1002/dev.420050403

[4] Povinelli DJ, Landau KR, Perilloux HK 1996 Self-recognition in young children using delayed versus live feedback: evidence of a developmental asynchrony. Child Dev. 67, 1540–1554. (doi:10.2307/1131717) 8890499

[5] Suddendorf T 1999 Children's understanding of the relation between delayed video representation and current reality: a test for self-awareness? J. Exp. Child Psychol. 72, 157–176. (doi:10.1006/jecp.1998.2485) 10047437 10.1006/jecp.1998.2485

[6] Miyazaki M, Hiraki K 2006 Delayed intermodal contingency affects young children's recognition of their current self. Child Dev. 77, 736–750. (doi:10.1111/j.1467-8624.2006.00900.x) 16686798 10.1111/j.1467-8624.2006.00900.x

[7] Skouteris H, Boscaglia L, Searl K 2009 Delayed self-recognition in 2.5-year-old children: evidence of a restricted sense of self? Eur. J. Dev. Psychol. 6, 258–280. (doi:10.1080/17405620701269367)

[8] Povinelli DJ 1995 The unduplicated self. In The self in infancy: theory and research (ed. Rochat P), pp. 161–192. Amsterdam, The Netherlands: Elsevier.

[9] Zelazo PD, Sommerville JA, Nichols S 1999 Age-related changes in children's use of external representations. Dev. Psychol 35, 1059–1071. (doi:10.1037/0012-1649.35.4.1059) 10442874 10.1037//0012-1649.35.4.1059

[10] Suddendorf T, Simcock G, Nielsen M 2007 Visual self-recognition in mirrors and live videos: evidence for a developmental asynchrony. Cogn. Dev. 22, 185–196. (doi:10.1016/j.cogdev.2006.09.003)

[11] Anderson JR, Gallup GG Jr 2011 Which primates recognize themselves in mirrors? PLoS Biol. 9, e1001024 (doi:10.1371/journal.pbio.1001024) 21390247 10.1371/journal.pbio.1001024 PMC3046971

[12] Bard KA, Todd BK, Bernier C, Love J, Leavens DA 2006 Self-awareness in human and chimpanzee infants: what is measured and what is meant by the mark and mirror test? Infancy 9, 191–219. (doi:10.1207/s15327078in0902_6)

[13] Hirata S 2007 A note on the responses of chimpanzees (*Pan troglodytes*) to live self-images on television monitors. Behav. Proc. 75, 85–90. (doi:10.1016/j.beproc.2007.01.005) 10.1016/j.beproc.2007.01.005 17324534

[14] Lin AC, Bard K, Anderson JR 1992 Development of self-recognition in chimpanzees (*Pan troglodytes*). J. Comp. Psychol. 106, 120–127. (doi:10.1037/0735-7036.106.2.120) 1600719 10.1037/0735-7036.106.2.120

[15] Povinelli DJ, Rulf AB, Landau KR, Bierschwale DT 1993 Self-recognition in chimpanzees (*Pan troglodytes*): distribution, ontogeny, and patterns of emergence. J. Comp. Psychol. 107, 347–372. (doi:10.1037/0735-7036.107.4.347) 8112048 10.1037/0735-7036.107.4.347

[16] Swartz KB, Evans S 1991 Not all chimpanzees (*Pan troglodytes*) show self-recognition. Primates 32, 483–496. (doi:10.1007/BF02381939)

[17] de Veer MW, Gallup GG, Theall LA, van den Bos R, Povinelli DJ 2003 An 8-year longitudinal study of mirror self-recognition in chimpanzees (*Pan troglodytes*). Neuropsychology 41, 229–234. (doi:10.1016/S0028-3932(02)00153-7) 10.1016/s0028-3932(02)00153-7 12459221

[18] Reiss D, Marino L 2001 Mirror self-recognition in the bottlenose dolphin: a case of cognitive convergence. Proc. Natl Acad. Sci. USA 98, 5937–5942. (doi:10.1073/pnas.101086398) 11331768 10.1073/pnas.101086398 PMC33317

[19] Plotnik JM, de Waal FBM, Reiss D 2006 Self-recognition in an Asian elephant. Proc. Natl Acad. Sci. USA 103, 17 053–17 057. (doi:10.1073/pnas.0608062103) 10.1073/pnas.0608062103 PMC1636577 17075063

[20] Anderson JR, Kuroshima H, Paukner A, Fujita K 2009 Capuchin monkeys (*Cebus apella*) respond to video images of themselves. Anim. Cogn. 12, 55–62. (doi:10.1007/s10071-008-0170-3) 18574604 10.1007/s10071-008-0170-3 PMC3639483

[21] Law LE, Lock AJ 1994 Do gorillas recognize themselves on television? In Self-Awareness in animals and humans: developmental perspectives (eds Parker ST, Mitchell RW, Boccia ML), pp. 308–312. Cambridge, UK: Cambridge University Press.

[22] Kaneko T, Tomonaga M. 2011 The perception of self-agency in chimpanzees (*Pan troglodytes*). Proc. R. Soc. B 278, 3694–3702. (doi:10.1098/rspb.2011.0611) 10.1098/rspb.2011.0611 PMC3203506 21543355

[23] Idani G, Hirata S 2006 Studies at the Great Ape Research Institute. In Primate perspectives on behavior and cognition (ed. Washburn DA) pp. 29–36. Washington, DC: American Psychological Association.

[24] Kärtner J, Keller H, Chaudhary N, Yovsi RD 2012 The development of mirror self-recognition in different sociocultural contexts. Monogr. Soc. Res. Child Dev. 77, 1–101. (doi:10.1111/j.1540-5834.2012.00689.x) 23153268 10.1111/j.1540-5834.2012.00688.x

[25] Paukner A, Anderson JR, Fujita K 2004 Reactions of capuchin monkeys (*Cebus apella*) to multiple mirrors. Behav. Process. 66, 1–6. (doi:10.1016/j.beproc.2003.11.001) 10.1016/j.beproc.2003.11.001 15062965

[26] Broesch T, Callaghan T, Henrich J, Murphy C, Rochat P 2011 Cultural variations in children's mirror self-recognition. J. Cross-Cult. Psychol. 42, 1018–1029. (doi:10.1177/0022022110381114)

[27] Matsuzawa T, Tomonaga M, Tanaka M (eds) 2006 Cognitive development in chimpanzees. Tokyo, Japan: Springer.

[28] Suddendorf T, Corballis MC 1997 Mental time travel and the evolution of the human mind. Gen. Soc. Gen. Psychol. Monogr. 123, 133–167. 9204544

[29] Roma PG, Silberberg A., Huntsberry ME, Christensen CJ, Ruggiero AM, Suomi SJ 2007 Mark tests for mirror self-recognition in capuchin monkeys (*Cebus apella*) trained to touch marks. Am. J. Primatol. 69, 989–1000. (doi:10.1002/ajp.20404) 17253635 10.1002/ajp.20404

[30] Hirata S, Fuwa K, Myowa M 2017 Data from: Chimpanzees recognize their own delayed self-image. Dryad Digital Repository. (http://dx.doi.org/10.5061/dryad.2g5r0) 10.1098/rsos.170370 PMC5579101 28878955

